# Nutritional risk index as a predictor of mortality in acutely decompensated heart failure

**DOI:** 10.1371/journal.pone.0209088

**Published:** 2018-12-14

**Authors:** Jae Yeong Cho, Kye Hun Kim, Hyun-Jai Cho, Hae-Young Lee, Jin-Oh Choi, Eun-Seok Jeon, Sang Eun Lee, Min-Seok Kim, Jae-Joong Kim, Kyung-Kuk Hwang, Shung Chull Chae, Sang Hong Baek, Seok-Min Kang, Dong-Ju Choi, Byung-Su Yoo, Youngkeun Ahn, Hyun-Young Park, Myeong-Chan Cho, Byung-Hee Oh

**Affiliations:** 1 Department of Cardiovascular Medicine, Chonnam National University Hospital, Gwangju, Korea; 2 Department of Internal Medicine, Seoul National University Hospital, Seoul, Korea; 3 Sungkyunkwan University College of Medicine, Seoul, Korea; 4 University of Ulsan College of Medicine, Seoul, Korea; 5 Chungbuk National University College of Medicine, Cheongju, Korea; 6 Kyungpook National University College of Medicine, Daegu, Korea; 7 The Catholic University of Korea, Seoul, Korea; 8 Yonsei University College of Medicine, Seoul, Korea; 9 Seoul National University Bundang Hospital, Seongnam, Korea; 10 Yonsei University Wonju College of Medicine, Wonju, Korea; 11 National Institute of Health (NIH), Osong, Korea; Kaohsiung Medical University Hospital, TAIWAN

## Abstract

**Background:**

We investigated the role of nutritional risk index (NRI) in predicting 1-year mortality in patients with acute decompensated heart failure (ADHF).

**Methods:**

Among 5,625 cohort patients enrolled in Korean Acute Heart Failure (KorAHF) Registry, a total of 5,265 patients who were possible to calculate NRI [NRI = (1.519 x serum albumin [g/dl]) + (41.7 x weight [kg]/ideal body weight [kg])] were enrolled. The patients were divided into 4 groups according to the NRI quartile; Q1 <89 (n = 1121, 69.9 ± 14.5 years, 632 males), Q2 89–95 (n = 1234, 69.7 ± 14.4 years, 677 males), Q3 95–100 (n = 1199, 68.8 ± 14.0 years, 849 males), Q4 >100 (n = 1711, 65.6 ± 14.5 years, 779 males). Primary end-point was all-cause mortality at 1-year clinical follow-up.

**Results:**

The 1-year mortality was significantly increased as the NRI quartile decreased, and the lowest NRI quartile was associated with the highest 1-year mortality (Q1: 27.5% vs. Q2: 20.9% vs. Q3: 12.9% vs. Q4: 8.7%, linear p <0.001). On Kaplan-Meier survival analysis, the significant inter-quartile difference was observed (p <0.001 for all). In multivariate analysis using Cox proportional hazard regression, the lowest NRI quartile was an independent predictor of 1-year mortality in patients with ADHF.

**Conclusions:**

Poor nutritional status as assessed by NRI and quartile grading of NRI was associated with 1-year mortality in Korean patients with ADHF. The assessment of nutritional status by NRI may provide additional prognostic information and thus would be useful in the risk stratification of the patients with ADHF.

## Introduction

Nutrition has been an essential risk factor in various cardiovascular diseases. There are numbers of evidences that nutritional status affects development of heart failure (HF) as well as its clinical outcomes. Malnutrition may result in cardiac remodeling and worsening of HF by promoting the production of catabolic cytokines, such as TNF-α, interleukin-6 and interlukin-1β that may lead to inflammation [1, 2]. Vice versa, an imbalance between anabolic and catabolic processes in patients with HF can cause malnutrition [3]. Despite these close association between nutritional status and HF, there is a difficulty in the diagnosis of malnutrition and cachexia in patients with HF because of body weight gain due to edema or excessive extracellular fluid in this clinical setting.[4]

The previous studies have shown that albumin or body mass index (BMI) which can reflect a nutritional status is associated with adverse clinical outcomes in various cardiovascular diseases [5–9]. However, the evaluation of nutritional status by single parameter such as albumin or BMI may not reflect nutritional status of the patients precisely. To overcome the limitations of single parameter approach, several indices or scoring systems were proposed and have been used to evaluate nutritional status. Nutritional risk index (NRI) is a commonly used method to evaluate the nutritional status of the elderly [10], and controlling nutritional status or Ulibarri's method (CONUT) score which incorporate serum albumin, cholesterol levels, and lymphocyte counts is also a useful tool for evaluating nutritional status [11]. Some studies evaluated the usefulness of CONUT score in patients with HF and demonstrated that CONUT score can predict poor outcome in terms of HF and non-HF readmissions, independently of body mass index (BMI) [12] and is useful for predicting the long-term prognosis of hospitalized patients with HF, especially in elderly population [13, 14]. However, the role of NRI has been poorly evaluated in a cohort of patients with acute decompensated heart failure (ADHF). Therefore, the aim of this study was to investigate the impacts of NRI on long-term clinical outcomes in patients with ADHF. The usefulness of NRI in predicting 1-year mortality in patients with ADHF was also compared to that of CONUT score.

## Materials and methods

### Study population

A total of 5,625 consecutive hospitalized patients for ADHF from 10 tertiary university hospitals were enrolled in Korean Acute Heart Failure (KorAHF) from March 2011 to February 2014 with a planned follow-up period through 2016. Patients who have signs or symptoms of HF and one of the following criteria are eligible for the study: (i) lung congestion or (ii) objective findings of LV systolic dysfunction or structural heart disease. Lung congestion has been defined as ‘congestion’ on a chest X-ray or as rales on physical examination. There are no exclusion criteria. The patients were classified into de novo (new-onset acute HF in a patient without previously known cardiac dysfunction), acute decompensation of chronic HF, or five clinical profiles (acute decompensated HF, hypertensive HF, pulmonary edema, cardiogenic shock, and right HF) by the attending physician according to the 2005 European Society of Cardiology (ESC) guidelines.[15] The class of high-output HF was not recorded. The study protocol was approved by the ethics committee at each hospital: Chonnam National University Hospital; Seoul National University Hospital; Samsung Medical Center; Asan Medical Center; Chungbuk National University hospital; Kyungpook National University hospital; Catholic University St. Mary’s Hospital; Yonsei University Severance Hospital; Seoul National University Bundang Hospital; Wonju Christian Hospital.

**Fig 1:**
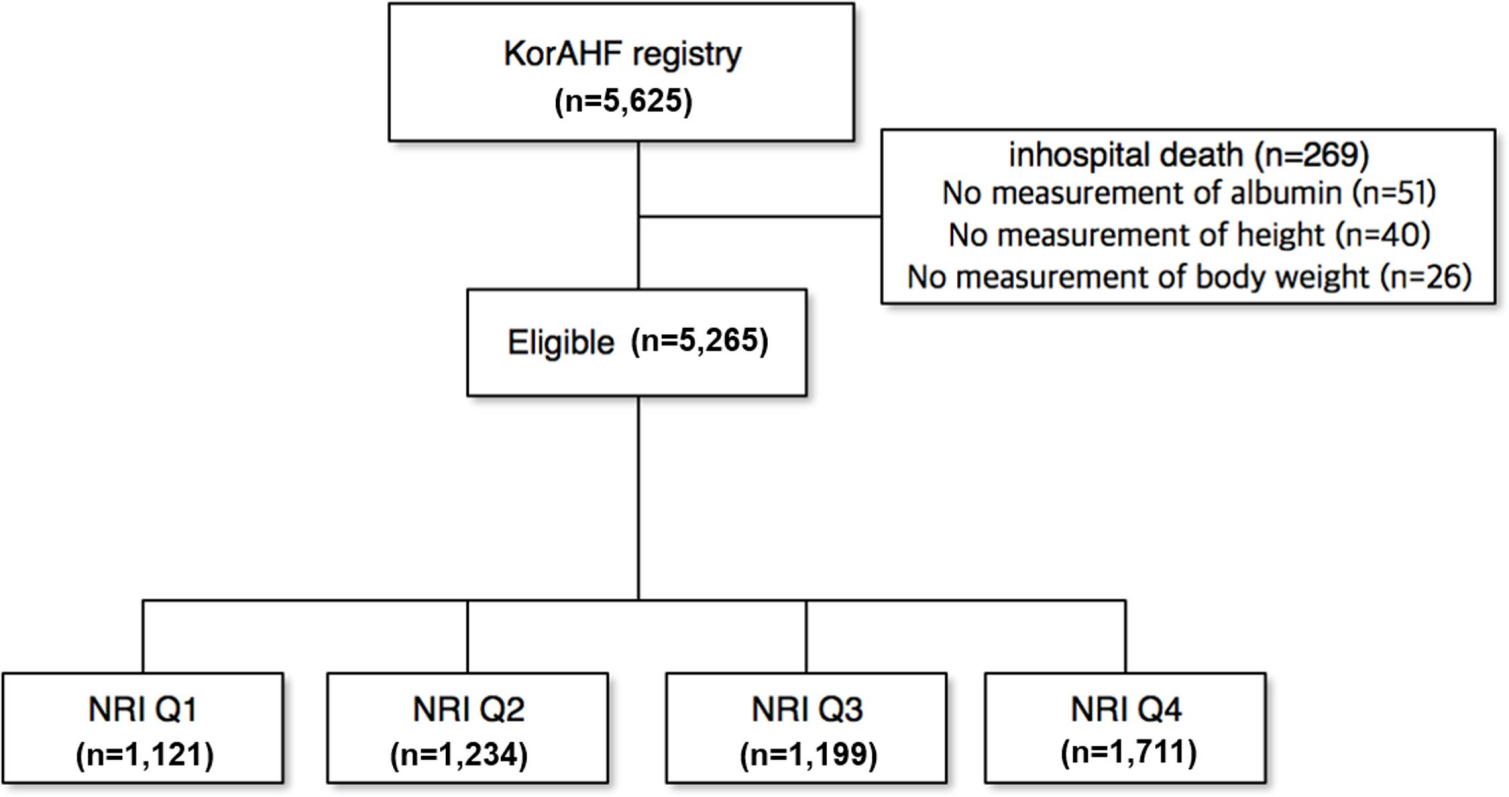
Study flow. KorAHF = Korea acute heart failure; NRI = nutritional risk index.

Among 5,625 ADHF cohort patients, a total of 5,265 patients who were possible to calculate NRI were incorporated into analysis. The patients were divided into 4 groups according to NRI quartile; Q1 <89 (n = 1121, 69.9 ± 14.5 years, 632 males), Q2 89–95 (n = 1234, 69.7 ± 14.4 years, 677 males), Q3 95–100 (n = 1199, 68.8 ± 14.0 years, 849 males), Q4 >100 (n = 1711, 65.6 ± 14.5 years, 779 males) (Fig 1). Primary endpoint was all-cause mortality and secondary endpoint was rehospitalization at 1-year clinical follow-up. Composite endpoint was all-cause mortality and rehospitalization at 1-year clinical follow-up.

### Data collection

As described in detail previously[16], written informed consents were obtained from all patients. If patients were unable to give consent due to disease severity, informed consent was obtained from a relative or legal representative. The attending physician completed a web-based case report form in the Clinical Data Management System (iCReaT) from the Korea National Institute of Health (NIH) with the assistance of a clinical research coordinator. The detailed variables and values collected at baseline admission and case definition are described in elsewhere.[16] If the patients were admitted via an emergency department (ED), the initial presentation and laboratory results at the ED are included in the baseline data. Echocardiographic exam was performed during the first few days of admission. After discharge, events including all-cause death, death from HF aggravation, and re-hospitalization for HF aggravation are recorded. The latest information on a patient’s clinical manifestation, biochemistry, and medication is collected at the first re-visit in 30 days, and at 3, 6, 12, 24, 36, 48, and 60 months. The follow-up data were collected from the patients by the attending physician and stored in the web-based case report form. The outcome data for subjects who had not been followed up have been ascertained by a telephone interview. In addition, the outcome data for patients lost to follow-up will be collected from the National Death Records.

### Definitions

Malnutrition was defined as the lowest quartile of NRI. NRI was calculated as: NRI = (1.519 x serum albumin [in g/dl] + (41.7 x weight [in kg] / ideal body weight [in kg]). The ideal body weight (IBW) was calculated was the Devine formula for men (IBW [kg] = 50 kg + 2.3 kg for each inch of height > 5 feet) and the Robinson formula for women (IBW [kg] 48.67 kg + 1.65 kg for each inch of height over 5 feet).[17] BMI was defined as weight in kilograms divided by height in meters squared (kg/m^2^) and obesity was defined as a BMI of 30.0.

CONUT score and Prognostic Nutritional Index (PNI) were also used to evaluate the nutritional status in patients with HF. CONUT score system was developed for hospitalized patients[11]. It thus enables evaluation of the protein reserves, calorie depletion, and immune defenses, respectively (Table 1). We classified the patients according to the CONUT score as: normal nutritional status (CONUT 0–1 points), mild malnutrition (CONUT score 2–4 points) and moderate to severe malnutrition (CONUT score ≥ 4 points). PNI was calculated as 10 x albumin level (g/dL) + 0.005 x lymphocyte count (/uL).

**Table 1:** Comparison of baseline characteristics according to NRI quartiles.

Variables	All (n = 5,265)	Quartile 1 NRI <89 (n = 1,121)	Quartile 2 NRI 89–95 (n = 1,234)	Quartile 3 NRI 95–100 (n = 1,199)	Quartile 4 NRI >100 (n = 1,711)	P for trend
Age (years)	68.4±14.5	69.9±14.5	69.7±14.4	68.8±14.0	65.6±14.5	<0.001
Male sex (n, %)	2782 (52.8)	632 (49.8)	677 (52.3)	849 (54.1)	779 (55.7)	0.001
Height (cm)	160.4±9.6	159.9±9.1	159.9±9.8	160.7±9.5	161.1±9.6	0.002
Weight (kg)	60.4±13.1	54.2±12.2	58.6±12.1	62.8±12.4	64.9±12.7	<0.001
Body mass index (kg/m2)	23.4±3.9	21.1±3.9	22.8±3.6	24.2±3.6	24.9±3.5	<0.001
Body surface area (m2)	1.6±0.2	1.6±0.2	1.6±0.2	1.7±0.2	1.7±0.2	<0.001
NYHA class III-IV	4706 (85.1)	1117 (88.1)	1128 (87.1)	1324 (84.3)	1137 (81.3)	<0.001
Ischemic etiology	2077 (37.6)	516 (40.7)	483 (37.3)	588 (37.5)	490 (35.1)	0.005
Systolic blood pressure (mmHg)	132.0±30.2	128.5±31.0	129.3±30.4	132.4±29.3	134.1±30.6	<0.001
Diastolic blood pressure (mmHg)	79.1±18.6	76.6±18.6	77.5±18.5	79.3±19.2	80.8±18.5	<0.001
Heart rate (bpm)	92.5±25.9	93.4±25.2	91.9±26.2	93.2±26.8	91.9±25.6	0.261
NRI (points)	95.6±8.7	82.8±5.3	92.0±1.7	97.6±1.6	104.3±3.1	<0.001
CONUT score (points)	2.5±2.0	4.6±2.1	2.6±1.6	1.8±1.2	1.5±1.5	<0.001
PNI (points)	45.3±7.2	39.2±7.3	43.7±5.6	46.7±4.4	52.3±5.1	<0.001
Hypertension	3109 (59.1)	735 (58.0)	741 (57.2)	955 (60.8)	839 (60.0)	0.099
Diabetes mellitus	1861 (35.3)	466 (36.8)	431 (33.3)	595 (37.9)	466 (33.3)	0.362
Dyslipidemia	2125 (40.4)	438 (38.3)	462 (39.6)	651 (45.7)	617 (48.6)	<0.001
Smoking history	2030 (38.6)	460 (36.3)	488 (37.7)	623 (39.7)	566 (40.5)	0.014
Alcohol history	2023 (38.4)	434 (34.2)	450 (34.7)	608 (38.7)	630 (45.1)	<0.001
Obesity	273 (5.2)	29 (2.3)	51 (3.9)	101 (6.4)	100 (7.2)	<0.001
Cerebrovascular disease	784 (14.9)	215 (17.0)	199 (15.4)	237 (15.1)	181 (12.9)	0.005
Ischemic heart disease	1461 (27.7)	371 (29.3)	370 (28.6)	473 (30.1)	346 (24.8)	0.030
Chronic kidney disease	734 (13.9)	253 (20.0)	207 (16.0)	214 (13.6)	115 (8.2)	<0.001
Atrial fibrillation	1456 (27.7)	291 (22.9)	370 (28.6)	453 (28.9)	405 (29.0)	0.001

Values are mean±SD. NRI, nutritional risk index.

### Statistical analysis

Continuous variables with normal distributions are presented as mean ± standard deviation and were compared using the student’s *t*-test or Mann-Whitney *U* test when group distributions were skewed. Categorical variables were compared using the chi-square test or Fisher’s exact test, where appropriate. The comparison of baseline characteristics and echocardiographic findings according to NRI quartile were performed using one-way analysis of variance. ROC-curve analysis was performed to compare NRI with CONUT score. COX proportional hazard regression was used to determine the independent predictors of 1-year mortality in patients with ADHF. Variables with p <0.1 on univariate regression analysis and clinically relevant variables were analyzed. All statistical tests were two-tailed and *p* value <0.05 were considered significant. Statistical analyses were performed using the Statistical Package for Social Sciences, version 18.0 (SPSS, PC version, Chicago, Illinois) and the comparisons of ROC curves were performed by use of MedCalc, version 16.8.4 (MedCalc Software bvba, Ostend, Belgium).

## Results and discussion

### Baseline characteristics of study population

Malnutrition in ADHF patients was not uncommon and the total number of the lowest NRI quartile was 1,121 out of 5,265 (21.3%). Demographic characteristics of NRI quartiles are summarized in Table 1. Patients with lower NRI quartiles were older and more likely to be female. BMI and body surface area were higher in the highest NRI quartile. Baseline heart rate, history of hypertension, and diabetes were not different among groups. Patients with the highest NRI quartile had the most frequent history of dyslipidemia, smoking history, and alcohol.

### Laboratory findings and echocardiographic findings

Laboratory findings among NRI quartiles are summarized in Table 2. Patients with the lowest NRI quartile had lower sodium concentration and hemoglobin level, but higher serum blood urea nitrogen and creatinine with lower glomerular filtration rate. Lipid profiles were all highest in patients with the highest NRI quartile. NT-proBNP had inverse relationship with NRI quartiles and the highest level of high-sensitivity C-reactive protein was noted in the lowest NRI quartile.

**Table 2:** Laboratory findings according to NRI quartiles.

Variables	All (n = 5,265)	Quartile 1 NRI <89 (n = 1,121)	Quartile 2 NRI 89–95 (n = 1,234)	Quartile 3 NRI 95–100 (n = 1,199)	Quartile 4 NRI >100 (n = 1,711)	P for trend
QRS duration (msec)	106.3±28.8	102.8±25.8	106.8±29.8	108.4±30.9	108.7±28.6	<0.001
White blood cell (/uL)	8568.6±4001.6	9001.1±4845.3	8560.2±3821.5	8447.2±3875.6	8724.2±3783.5	0.003
Sodium (mEq/L)	137.6±4.7	136.4±5.3	136.8±5.1	138.0±4.5	138.7±4.1	<0.001
Hemoglobin (g/dL)	12.4±2.3	11.4±2.2	12.1±2.2	12.6±2.2	13.5±2.1	<0.001
Platelet (x1000/uL)	211.2±86.2	217.1±98.6	207.8±87.7	206.5±91.8	210.6±76.8	0.010
Blood urea nitrogen (mg/dl)	25.8±15.9	29.5±19.0	27.9±16.9	25.8±16.4	22.2±12.4	<0.001
Creatinine (mg/dl)	1.5±1.5	1.7±1.9	1.5±1.5	1.4±1.3	1.3±1.1	<0.001
GFR (ml/min)	73.8±39.3	69.0±43.2	70.2±37.8	72.5±37.3	79.9±38.1	<0.001
CK-MB (ng/mL)	8.0±27.4	9.4±29.7	10.4±56.5	8.4±28.8	9.9±44.1	0.620
Cardiac Troponin-I (ng/mL)	2.3±17.8	4.1±27.1	2.5±18.6	2.6±16.7	2.3±16.1	0.148
Glucose (mg/dl)	154.3±76.0	161.5±83.5	157.1±79.5	153.7±75.3	150.8±69.8	0.003
Hb A1c (%)	6.8±1.4	6.9±1.5	6.8±1.4	6.7±1.4	6.6±1.2	0.015
Total Cholesterol (mg/dl)	152.2±42.9	142.9±46.5	145.1±40.7	152.0±39.5	164.9±43.0	<0.001
Triglycerides (mg/dl)	99.4±59.1	93.3±53.6	90.1±48.4	98.0±58.4	112.3±68.3	<0.001
HDL cholesterol (mg/dl)	41.7±13.8	37.7±14.5	41.6±15.4	41.6±13.0	43.9±12.7	<0.001
LDL cholesterol (mg/dl)	93.8±37.0	89.4±39.5	88.1±33.8	93.2±34.8	100.6±39.1	<0.001
NT-proBNP (pg/ml)	4736 (2086–11313)	10519 (4040–23750)	6477 (3131–13633)	4085 (1971–9511)	2844 (1273–5865)	<0.001
hsCRP (mg/dl)	0.65 (0.20–2.28)	1.83 (0.53–5.48)	0.85 (0.30–3.02)	0.54 (0.18–1.65)	0.30 (0.11–0.92)	<0.001

Values are mean±SD. NRI, nutritional risk index; GFR, glomerular filtration rate; CK, Creatinine Kinase; Hb, hemoglobin; HDL, high-density lipoprotein; LDL, Low-density lipoprotein; NT-proBNP, N-terminal pro B-type natriuretic peptide; hsCRP, high-sensitivity C-reactive protein

Echocardiographic findings according to NRI quartiles are summarized in Table 3. LV diastolic and systolic chamber size and LVEF were higher in the highest NRI quartile, possibly reflecting lower volume status in the lowest NRI quartile. Patients in the highest NRI quartile also showed higher e’ and thus lower E/e’ and lower systolic pulmonary artery pressure reflecting better LV diastolic function. Among those variables mentioned above, NRI had a correlation between LVEF (r = 0.031, p = 0.026), LVEDD (r = -0.113, <0.001), NT-proBNP r = -0.301, <0.001) and hsCRP (r = -0.283, <0.001), which were not so strong.

**Table 3:** Echocardiographic findings according to NRI quartiles.

Variables	All (n = 5,265)	Quartile 1 NRI <89 (n = 1,121)	Quartile 2 NRI 89–95 (n = 1,234)	Quartile 3 NRI 95–100 (n = 1,199)	Quartile 4 NRI >100 (n = 1,711)	P for trend
LVEF (%)	38.1±15.6	37.5±15.5	37.0±15.5	37.9±15.6	38.8±15.8	0.022
LVEDD (mm)	57.4±10.0	55.3±9.9	57.8±10.2	57.9±10.1	58.2±9.9	<0.001
LVESD (mm)	45.1±12.4	43.6±11.9	45.6±12.6	45.5±12.4	45.4±12.5	<0.001
Left atrial diameter (mm)	48.3±9.8	46.0±9.9	48.6±9.8	49.2±9.3	48.5±10.2	<0.001
E velocity (m/s)	0.94±0.39	0.91±0.37	0.98±0.39	0.97±0.42	0.90±0.36	<0.001
e’ velocity (cm/s)	5.0±2.3	4.9±2.1	4.9±2.2	5.0±2.1	5.2±2.8	0.005
E/e’	21.2±11.4	21.3±11.4	22.4±12.1	21.7±12.0	19.5±10.1	<0.001
Systolic pulmonary artery pressure (mmHg)	43.8±15.1	45.2±14.3	45.5±15.3	44.6±15.5	40.8±14.5	<0.001

Values are mean±SD. NRI, nutritional risk index; LVEF, left ventricular ejection fraction; LVEDD, left ventricular end diastolic dimension; LVESD, left ventricular end systolic dimension; E, early diastolic velocity of mitral inflow; e’, early diastolic mitral annular velocity

### Medical treatment according to the NRI quartiles

The comparison of discharge medications according to the NRI quartiles are summarized in Table 4. Patients in the lowest NRI quartile used ACE inhibitor, ARB, and beta-blockers least. However, nitrate was most frequently used in the lowest NRI quartile. Guideline-based HF treatment, such as ACE inhibitor, ARB, or beta-blockers was most infrequently used in the lowest NRI quartile. Statin was used most frequently in the highest NRI quartile. Amiodarone was used more commonly in the higher NRI quartile, but this showed no significant statistical difference. Digoxin tended to be used in the lower NRI quartile despite lower incidence of atrial fibrillation in the lowest NRI quartile (Table 1).

**Table 4:** Comparison of medications at discharge according to NRI quartiles.

Variables	All (n = 5,265)	Quartile 1 NRI <89 (n = 1,121)	Quartile 2 NRI 89–95 (n = 1,234)	Quartile 3 NRI 95–100 (n = 1,199)	Quartile 4 NRI >100 (n = 1,711)	P for trend
ACE inhibitor	1560 (29.6)	323 (25.5)	386 (29.8)	416 (26.5)	449 (32.1)	0.003
Angiotensin receptor blocker	2091 (39.7)	432 (34.1)	452 (34.9)	648 (41.3)	570 (40.8)	<0.001
Beta blocker	2749 (52.2)	572 (45.1)	638 (49.3)	809 (51.5)	742 (53.1)	<0.001
Nitrate	1192 (22.6)	322 (25.4)	328 (25.3)	329 (21.0)	238 (17.0)	<0.001
Furosemide	3829 (72.7)	837 (66.0)	954 (73.7)	1144 (72.9)	989 (70.7)	0.019
Amiodarone	381 (7.2)	94 (7.4)	93 (7.2)	120 (7.6)	114 (8.2)	0.402
Digoxin	1379 (26.2)	330 (26.0)	353 (27.3)	411 (26.2)	319 (22.8)	0.042
Warfarin	1557 (29.6)	261 (20.6)	383 (29.6)	486 (31.0)	438 (31.3)	<0.001
Aspirin	2901 (55.1)	675 (53.2)	671 (51.8)	854 (54.4)	770 (55.1)	0.174
Statin	2247 (42.7)	459 (36.2)	497 (38.4)	684 (43.6)	649 (46.4)	<0.001
No use of beta blocker or RASI	3134 (59.5)	853 (67.3)	811 (62.6)	930 (59.2)	799 (57.2)	<0.001

Values are mean±SD. NRI, nutritional risk index; ACE, angiotensin converting enzyme; RASI, renin-angiotensin system inhibitor

### Clinical outcomes according to NRI quartiles

**Fig 2:**
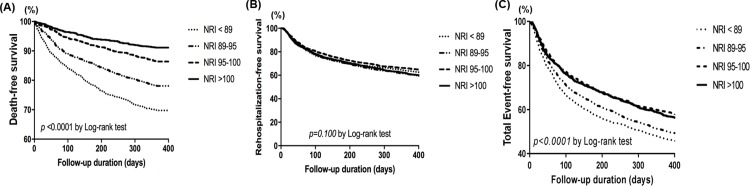
(A) Kaplan-Meier survival curves for 1-year mortality according to NRI quartiles. (B)Kaplan-Meier survival curves for rehospitalization-free survival according to NRI quartiles. (C)Kaplan-Meier survival curves for composite endpoint according to NRI quartiles. NRI = nutritional risk index.

There were significant differences in one-year mortality among NRI quartiles (from the lowest to highest quartile: 26.8% vs. 21.8% vs. 13.9% vs. 9.1%, linear p <0.001). Kaplan-Meier survival curves showed the highest 1-year mortality in patients with the lowest NRI quartile (Fig 2A). However, rehospitalization-free survival was not different among NRI quartiles (from the lowest to highest quartile: 46.8% vs. 46.5% vs. 50.7% vs. 47.0%, p = 0.110) (Fig 2B). Composite endpoint was more frequently observed in lower NRI quartiles (NRI <95) (Fig 2C).

In multivariate analysis using COX proportional hazard regression showed the lowest NRI quartile (NRI <89) was an independent predictor of 1-year mortality in patients with ADHF (HR 1.84, 95% CI 1.50–2.27, p <0.001) (Table 5).

**Table 5:** Independent predictors for 1-year mortality in patients with acute heart failure.

Variables	Hazard ratio	95% CI	p value
Age >75 years	1.78	1.46–2.18	<0.001
NYHA Fc IV	1.24	0.92–1.67	0.150
Systolic blood pressure <100 mmHg	1.41	1.10–1.80	0.007
Renal dysfunction	1.48	1.19–1.83	<0.001
LVEF <30%	1.14	0.92–1.41	0.222
Na+ <135 mEq/L	1.63	1.34–1.99	<0.001
Hemoglobin <10 g/dL	1.12	0.878–1.43	0.365
Nutritional Risk Index <89	1.84	1.50–2.27	<0.001
Uric acid >7.5 mg/dL	1.12	0.91–1.38	0.280
High BNP level	1.38	1.13–1.68	<0.001
Hypertension	1.16	0.94–1.43	0.178
Diabetes mellitus	0.85	0.70–1.05	0.128
Smoking	1.13	0.92–1.37	0.246
QRS duration	1.00	1.00–1.01	0.079
E/e’	1.01	1.00–1.02	0.002
High-sensitivity C-reactive protein	1.02	1.00–1.04	0.090
No use of beta blocker or RASI	1.39	1.38–2.13	0.002

Values are mean±SD. NYHA, New York Heart Association; LVEF, left ventricular ejection fraction; BNP, brain natriuretic peptide; RASI, renin-angiotensin system inhibitor

**Fig 3:**
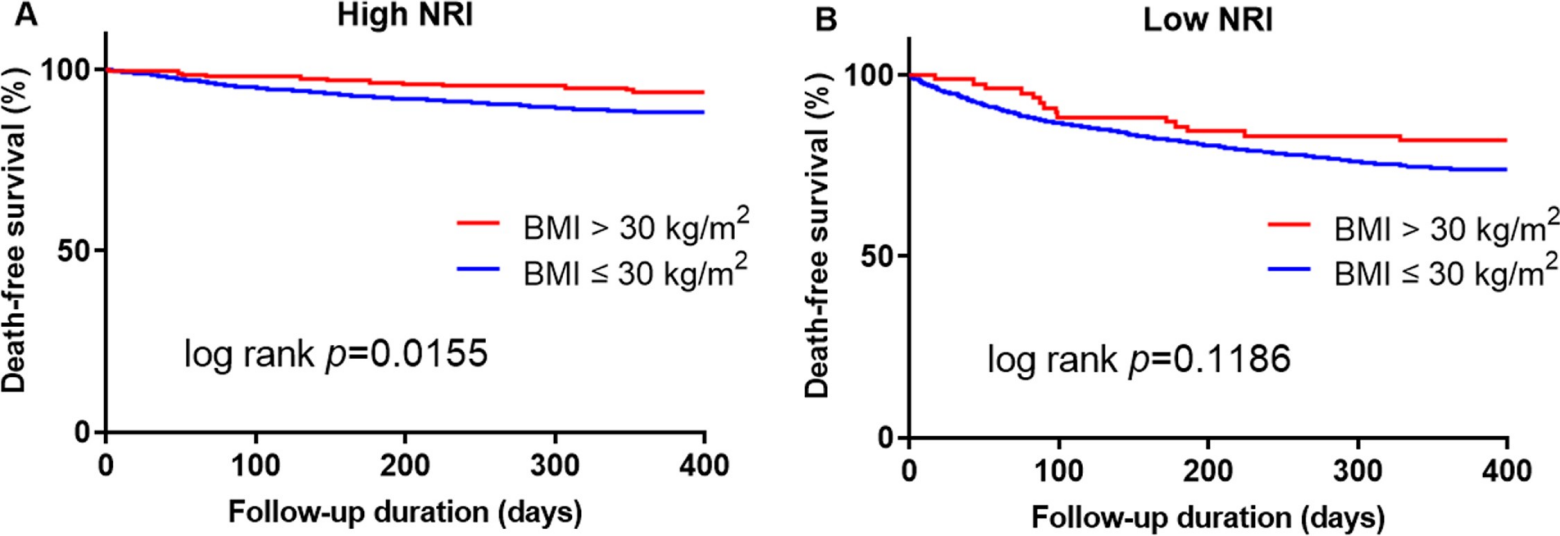
Comparison of death-free survival between patients with body mass index> 30 kg/m^2^ versus ≤ 30 kg/m^2^ in high nutritional risk index (A) and low nutritional risk index (B).

NRI showed different impact on risk stratification of obesity. In high NRI group (NRI ≥95), patients with BMI >30 kg/m^2^ showed better survival than those with BMI ≤30 kg/m^2^. However, there was no difference in low NRI group (NRI <95) (Fig 3).

### ROC curve analysis for 1-year mortality

**Fig 4:**
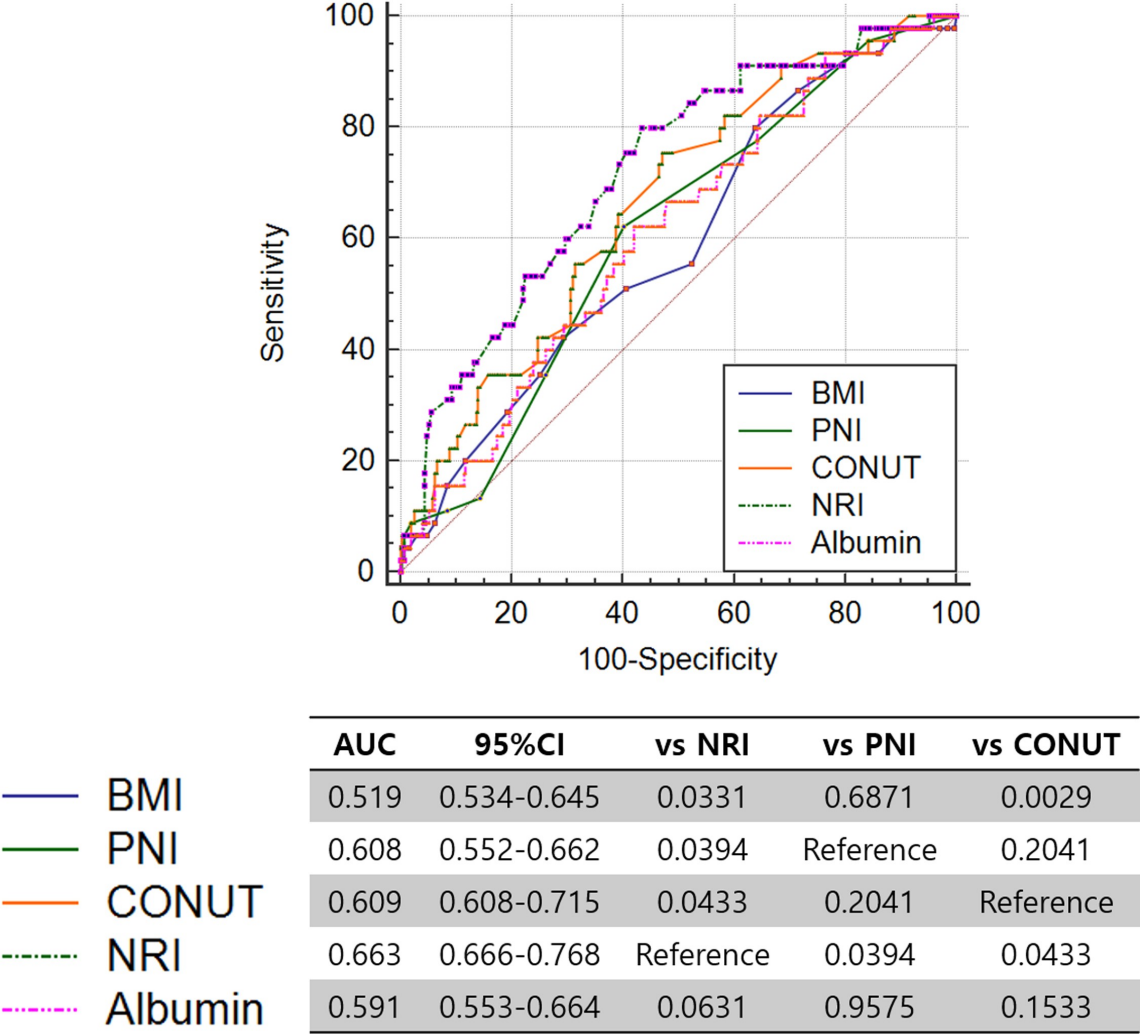
Receiver operating characteristics-curve analysis among variables associated with nutrition. CONUT = COntrolling NUTritional.

ROC-curve analysis showed that NRI (AUC = 0.653, p<0.001 vs. all) predicted mortality better than albumin only (AUC = 0.628), BMI (AUC = 0.611), or CONUT score (AUC = 0.594) in patients with ADHF (Fig 4).

NRI showed a sensitivity of 67.8% and specificity of 55.9% for prediction of 1-year mortality with the cut-off value of 96.24.

## Discussion

The present study investigated the impacts of nutritional status on clinical outcomes in patients with ADHF and demonstrated several important findings. First, poor nutritional status is not uncommon in patients with ADHF (NRI Q1 21.3%). Second, the lowest NRI quartile is associated with the highest 1-year mortality and 1-year mortality is significantly increased as the NRI quartile decreased in patients with ADHF. Third, ROC curve analysis suggested that NRI would be a better tool for evaluating nutritional status in patients with ADHF than any other variables such as albumin, BMI, or CONUT score. Fourth, NRI is an independent predictor of long-term mortality even after adjusting for a number of clinically relevant variables. To the best of our knowledge, this is the largest study that investigates the impacts of NRI on clinical outcomes in a large population of AHF cohort. Therefore, the present study suggested that the evaluation of nutritional status should be incorporated in the risk stratification of the patients with HF and NRI would be a simple and useful tool for this purpose.

### The role of nutritional status in risk scoring system: CONUT vs. NRI

In the present study, NRI predicted HF mortality as a single variable calculated only with serum albumin and body weight. Although NRI has such a simpler calculation than other detailed nutritional indices, such as Subjective Global Assessment [18] and Mini Nutritional Assessment [19] including dietary intake, weight change, and physical examination findings of muscle and fat, it has more powerful effect in predicting outcomes. NRI itself is a strong independent predictor for HF mortality even when adjusted by other important variables (Table 5). In addition, NRI in the present study showed better predictability for long-term mortality than other variables which reflect nutritional status such as albumin, BMI. Every risk scoring system for ADHF patients may need NRI in their scoring system. Although Seattle HF model has been a decent one for prediction of survival in HF[20], NRI components such as albumin and body weight were not included in the model. Modified TIMI risk index also applied to prediction of mortality at 120 days in AHF patients[21]. However, this score system required heart rate, age, and systolic BP, but not nutritional index. Incorporating NRI into those systems may strengthen the ability to predict prognosis in ADHF. Recently, CONUT score is being used in many studies[12, 22–24]. However, NRI was superior compared with CONUT score in the present study. The study by Narumi and colleagues had a smaller number of patients compared to our study (n = 388 vs. n = 5,265) and the predictive power was not compared between risk score system with ROC curve analysis. In multivariate analysis, p-value for CONUT score and NRI was same and the hazard ratio of NRI was relatively high as 6.0. Aggravation of HF may result in generalized edema and serum albumin level decreased in the setting of body fluid excess. These relationship of two components may increase the predictive value of NRI not only for nutritional status but also for HF status. This concept may be an explanation for the reason why NRI could predict HF mortality. CONUT score.

### Nutritional status and LV diastolic function

In the present study, e’, E/e’, and systolic pulmonary artery pressure were all better in the highest NRI quartile. Since diastolic dysfunction is not uncommon and important prognostic factor in patients with ADHF, this also should be illuminated. Wang et al. reported that bone mineral density was associated with LV diastolic function in women[25]. However, in a study of 31,334 apparently healthy Korean adults who underwent echocardiography, Park et al. reported that LV diastolic dysfunction was more frequent in patients with higher BMI[26]. Although higher NRI does not necessarily mean higher BMI or obesity, diastolic dysfunction and NRI in our result had positive correlation. Another explanation for this relationship would be sarcopenia. Sarcopenia, which is often accompanied by malnutrition, reportedly contributes to the muscle weakness and diastolic dysfunction in heart failure[27].

### The relationship of NRI and obesity in ADHF

Attempts have been made for the explanation of better clinical outcomes in HF patients with higher NRI quartile by obesity paradox. In the present study, obesity was most frequently observed in the highest NRI quartile (Table 1). Also, there was statistically significant linear trend of increased incidence of obesity according to increasing NRI score. Although there were only 5.4% of patients with obesity in total study population, indeed BMI was increased along higher NRI quartile. Even a patient does not meet obesity criteria, BMI and nutritional status may be collinear according to a study of 244 very old HF patients[28]. It has been reported that obesity itself may be protective in HF, because it may be a marker of nutrition and activate less tumor necrosis factor-alpha which worsens heart failure[29]. Obesity paradox in HF was demonstrated in numerous studies including that of Shah et al.[30] They concluded that a lower BMI was associated with age, disease activity, and a higher risk of death in ADHF. However, in subgroup analysis obesity paradox existed only in older age (>75 years), lower LV systolic function (LVEF <50%), non-diabetics, and de-novo HF. In the present study, obesity paradox was evident only in patients in higher NRI group (Fig 3). This indicate that NRI may give a clue for interpretation of obesity paradox in patients with HF.

### Study limitations

The present study has several limitations. First, this was not a randomized-controlled study. However, Cox proportional hazard method showed that NRI independently predicted 1-year mortality and our study is the largest prospective cohort that investigated the effect of NRI on post-discharge outcomes in AHF patients to our best knowledge. Second, the formula for calculating ideal body weight is not standardized to date. Third, the prognostic significance of NRI in chronic phase of HF has been already shown in a previous study [31]. Body weight is difficult to measure on admission and not always reliable because of peripheral edema. Therefore, body weight reflects body fluid volume rather than nutritional status in acute phase of HF. However, this difference of body weight is not so great and relatively similar to each other. Thus the impact on outcome may not be changed. Fourth, from the ROC curve analysis, AUC values of NRI and CONUT were 0.653 and 0.594, respectively, and both were not highly enough as prognostic markers. However, NRI were superior to CONUT score or PNI in this study population. Combination of more than two prognostic markers including NRI would be warranted in the future study so that we might overcome the limitation of low AUC level. Fifth, since we included only East Asians, we do not know whether the study results can be extrapolated to other ethnicities. Finally, due to the nature of the study design, the study results are at best hypothesis-generating, and the effect of nutritional intervention on clinical outcomes must be confirmed in further randomized-controlled clinical trials.

## Conclusions

Poor nutritional status as assessed by NRI and quartile grading of NRI was associated with 1-year mortality in Korean patients with ADHF. In addition, NRI was one of the independent predictors of 1-year mortality. The assessment of nutritional status by NRI may provide additional prognostic information and thus would be useful in the risk stratification of the patients with ADHF. NRI should be incorporated into novel risk scoring system for prediction of clinical outcomes in patients with ADHF. Furthermore, well designed randomized trial assessing the effect of nutritional intervention for HF patients would be warranted in the future.
